# Anti-Cancer Drug Sensitivity Assay with Quantitative Heterogeneity Testing Using Single-Cell Raman Spectroscopy

**DOI:** 10.3390/molecules23112903

**Published:** 2018-11-07

**Authors:** Yong Zhang, Jingjing Xu, Yuezhou Yu, Wenhao Shang, Anpei Ye

**Affiliations:** 1 Key Laboratory for the Physics and Chemistry of Nanodevices, School of Electronics Engineering and Computer Science, Peking University, No.5 Yiheyuan Road, Beijing 100871, China; zhangyong2006@pku.edu.cn; 2 Beijing Institute of Biomedicine, No.15 Xinjiangongmen Road, Beijing 100091, China; 3 Academy for Advanced Interdisciplinary Studies, Peking University, No.5 Yiheyuan Road, Beijing 100871, China; xu_jingjing@pku.edu.cn (J.X.); 13811636947@163.com (Y.Y.); wenhao_shang@pku.edu.cn (W.S.)

**Keywords:** raman spectroscopy, medical optics, biotechnology, drug sensitivity testing, cancer cells

## Abstract

A novel anti-cancer drug sensitivity testing (DST) approach was developed based on in vitro single-cell Raman spectrum intensity (RSI). Generally, the intensity of Raman spectra (RS) for a single living cell treated with drugs positively relates to the sensitivity of the cells to the drugs. In this study, five cancer cell lines (BGC 823, SGC 7901, MGC 803, AGS, and NCI-N87) were exposed to three cytotoxic compounds or to combinations of these compounds, and then they were evaluated for their responses with RSI. The results of RSI were consistent with conventional DST methods. The parametric correlation coefficient for the RSI and Methylthiazolyl tetrazolium assay (MTT) was 0.8558 ± 0.0850, and the coefficient of determination was calculated as R^2^ = 0.9529 ± 0.0355 for fitting the dose–response curve. Moreover, RSI data for NCI-N87 cells treated by trastuzumab, everolimus (cytostatic), and these drugs in combination demonstrated that the RSI method was suitable for testing the sensitivity of cytostatic drugs. Furthermore, a heterogeneity coefficient *H* was introduced for quantitative characterization of the heterogeneity of cancer cells treated by drugs. The largest possible variance between RSs of cancer cells were quantitatively obtained using eigenvalues of principal component analysis (PCA). The ratio of *H* between resistant cells and sensitive cells was greater than 1.5, which suggested the *H*-value was effective to describe the heterogeneity of cancer cells. Briefly, the RSI method might be a powerful tool for simple and rapid detection of the sensitivity of tumor cells to anti-cancer drugs and the heterogeneity of their responses to these drugs.

## 1. Introduction

Anti-tumor drug sensitivity testing (DST) is essential for cancer treatment, especially for individualized cancer therapy (ICT) [1,2,3]. Cancer cells from the same patients or from the same populations of cancer cell lines have significant differences, and this functional and phenotypic heterogeneity is virtually universal. In addition, patients respond uniquely despite the similarity of their cancer histological phenotypes. Drug responses in different treatment phases may vary for the same patient [4,5,6,7]. These variations may result in therapeutic failure or toxicity and patient death. Thus, cancer is difficult to cure due to this heterogeneity. ICT is a method directed against cancer heterogeneity, and DST provides an experimental basis of evaluating sensitivity or resistance to ICT in advance [1,8].

DST can be performed in vivo or in vitro by comparing the therapeutic efficacies of candidate drugs against surgically removed tumor to identify optimal drug selection. For in vivo DST, tumors are xenografted into immune-deficient mice, and drug treatments are applied and evaluated [9,10]. DST in vivo is labor intensive, expensive, and technically demanding, which limits its clinical application. DST in vitro is easier and more accessible. There are various sorts of drug sensitivity testing methods in vitro [3], and the MTT assay is commonly used to assess cell viability [11,12,13]. In vitro DST methods have limitations, including interference of normal cells and chemicals. Traditional DST is based on bulk experimentation which reflects the overall state of the cell samples, so intrasample heterogeneity cannot be quantified. Significant quantity changes of cancer cells are highly important in these measurements, so overlooking malignant tumor cells may cause crucial issues in cancer treatment. Also, targeted (cytostatic) anticancer drugs have fewer inhibitory effects on tumor proliferation compared to cytotoxic anticancer drugs, so conventional DST is unsuitable for evaluating targeted anticancer drugs [3]. Currently, sensitivity to targeted drugs is usually evaluated with molecular biological methods, such as gene mutation analysis [14], gene polymorphism analysis, and detection of gene expression [15]. A recent study also showed the potential of RS to monitor targeted anticancer drug resistance [16].

Single-cell Raman spectrum intensity (RSI) measurements for anti-cancer drug sensitivity assays could overcome the limitations mentioned above. Single-cell Raman spectroscopy (SCRS) can provide detailed quantitative information about individual living cells. It may be used to observe different cells and to detect cellular dynamic variation on the single cell level [17,18]. Previously, it was proposed that the therapeutic efficacy of conventional therapy, including chemotherapy, radiotherapy, etc., is affected by the number or the functions of cancer stem cells in tumors, because the cancer treatments induce the cancer stem cells to differentiate into drug resistant cells [3,19,20,21]. Because stem cells and their derivatives may be distinguished from cancer cells using Raman spectroscopy [22,23,24], combining this with drug sensitivity testing may present advantages over conventional DST. More importantly, the intensity of SCRS is positively correlated with cell survival state after drug treatment [25,26,27,28]. In addition, protein denaturation and DNA degradation related to cell death also can be evaluated by the variability of RSI measurements. Previous experiments have shown that all peaks of SCRS were significantly decreased after cells were treated with chemotherapeutic drugs compared to a control group, and the results obtained with the RSI method were consistent with traditional cytotoxicity assays [25]. Chemotherapeutic drugs typically induce oncosis, which will show up as decreased RS intensity due to cell swelling and its resulting lower density. Furthermore, cancer cell proliferation is decreased after molecularly-targeted drug treatment. The intracellular spatial distribution and metabolism of molecularly-targeted drug in different cancer cells can also be studied by using Raman imaging [29]. In our study, HER2 and mTOR were used as targets for estimating molecularly-targeted drug effects. HER2, a member of the human epidermal growth factor receptor family, is aberrantly expressed in certain malignancies, including breast and gastric cancer, primarily due to HER2 genomic amplification. In HER2-positive malignancies, this protein can stimulate downstream RAS-RAF-ERK and PI3K-PTEN-AKT signaling, and plays a key role in cell proliferation [30]. HER2 is the preferred therapeutic target in such cancers. Trastuzumab is an anti-HER2 monoclonal antibody and a standard therapy for HER2-positive cancers [31,32,33]. The mammalian target of rapamycin (mTOR) pathway is a central sensor for nutrient and energy availability. It regulates the cell cycle, growth, and proliferation through the PI3K-AKT-mTOR signaling pathway. Everolimus (RAD001) is a rapamycin derivative which inhibits the serine/threonine kinase activity of mTOR, and significantly inhibits tumor growth [34].

Furthermore, unlike the traditional DST method, SCRS measures individual cell responses to drugs. This has advantages for quantitative analysis of cellular heterogeneity. Cellular heterogeneity describes the observation that different tumor cells can show distinct morphological and phenotypic profiles, including cellular morphology, gene expression, metabolism, motility, proliferation, and metastatic potential. Using chemometrics, such as principal component analysis (PCA), the heterogeneous characteristics of cancer cells can be quantitatively evaluated after anti-cancer drug treatment. Moreover, RS is label-free and non-contacting, and it can identify cellular abnormal alteration in molecular structure and biochemical compositions prior to cellular morphological changes. RS is thus rapid, low-interference, and has minimal sample requirements.

In this study, we used RSI to assay human gastric carcinoma cells’ responses to chemotherapeutic drugs, and compared it to MTT to verify its stability and reproducibility in anti-cancer drug sensitivity assays. The parametric correlation coefficient of drug responses derived from MTT and RSI data were calculated with Pearson’s correlation coefficient, and a two-tailed Student’s *t*-test was used to assess statistical differences. Pharmacologically, the dose–response relationship describes the change in effect on an organism, tissue, or cells caused by differing levels of exposure (doses) to a drug after a certain exposure time [35], and dose–response curves are often fit to a classical Hill equation [36]. Here, we fit the relationship between drug dose and the cell response (RSI). The results indicated that our experimental data meet the dose–response relationship. Furthermore, we show that RSI can be used for sensitivity testing of targeted anti-cancer drugs. In brief, we propose a new method of quantifying heterogeneous characteristics of cancer cells by treating cells with drugs. This method supplements the existing DST method.

## 2. Materials and Methods

### 2.1. Confocal Raman Spectroscopy

We constructed an optical configuration for the back-scattering RS system (Figure S1 in the Supporting Information) as described previously [37,38]. In brief, a single continuous wave laser of 532 nm (Excelsior-532-200, Spectra-Physics, Santa Clara, CA, USA) with working power of 40 mW was coupled into an inverted microscope (Axiovert 200, Zeiss, Oberkochen, Germany). A 100 ×/1.25 oil objective (N-Achroplan, Zeiss, Oberkochen, Germany) with a working distance of 0.29 mm was used for RS measurement. Overall, considering the signal-to-noise ratios (SNR) of RS and photodamage to the cell, an optimal laser power of 20 mW on the cancer cell was used. The back-scattering Raman light was collected with the same objective and was directed into a spectrometer (SpectraPro2300i, 1200 g/mm blazed at 500 nm, Acton Research Co., Acton, MA, USA). A pinhole of 100 μm diameter was used to create a confocal system. A notch filter (532 nm, Thorlabs, Newton, NJ, USA) was placed before the entrance of the spectrometer to filter Rayleigh scattered light from the sample. Each RS was recorded with a high sensitivity liquid nitrogen-cooled spectroscopic CCD (Spec-10:400BR/LN, Princeton Instruments, Trenton, NJ, USA).

### 2.2. Cell Culture

Five human gastric carcinoma cell lines (BGC 823, SGC 7901, MGC 803, AGS, and NCI-N87) from Beijing Cancer Hospital were used. Prior to experiments, each cell line was placed in standard culture medium: RPMI-1640 medium (Macgene, Hangzhou, China) supplemented with 10% fetal bovine serum (Tianhang Biological Technology Co. Ltd., Beijing, China) without antibiotics and cultured at 37 °C with a relative humidity of 95% and 5% CO_2_. Cell lines were expanded (less than five passages) for RS measurements and cryopreserved to conserve the seed stocks.

### 2.3. Anti-Cancer Drug Treatment of Cancer Cells

Cancer cells from one passage were cultured, transferred to culture plates in the logarithmic growth phase, and incubated at 37 °C in 5% CO_2_ before RS measurements. After cells reached 50% confluence, drugs were added to the cancer cell plates, and then the drugs and cells were fully mixed by slightly swaying the cell plate. Control groups of cells were used with the same volume of culture medium (with RPMI-1640 medium and fetal bovine serum) and incubated together with experimental groups (drug treatment groups). Before RS measurements, 0.25% trypsin (Macgene, China) was added to remove cells. Then, 2 mL complete medium was added to terminate the digestion. Then, cells were transferred via pipette to PBS and transferred to a 15 mL centrifuge tube. Both control and drug-treated cells were washed with PBS three times by centrifugation at 1000 rpm (800× *g*). It should be noted that the dead and disrupted cells were removed during the wash, which ensures that all the cells for the RS measurement were alive. Supernatants were discarded after the wash, and post-centrifugation cells were diluted in 5 mL PBS and mixed well. Finally, 80 μL of each group was measured for RS measurement. For targeted drug treatment for a long duration, culture medium with targeted drugs was renewed every 48 h.

The molecularly-targeted anti-cancer drugs, trastuzumab and everolimus, were applied to human gastric cancer cells NCI-N87, which express HER2 and mTOR simultaneously. The drug effects were assayed using the RSI method.

### 2.4. RS Measurements

To collect RS signals with high SNR, a sample chamber was designed. A cleaned quartz coverslip (CFS-1010, UQG Ltd., Milton, Cambridge, UK, thickness 0.1 mm) was fixed at the bottom of the sample chamber (top diameter 8 mm; bottom diameter 6 mm, depth 2 mm). Another quartz coverslip was placed on top of the chamber during the experiment to prevent evaporation and contamination. Washed and rinsed cell samples diluted in PBS were placed into the sample chamber. To ensure consistent spectra collection for individual cells, the laser was focused on the cell center (nucleus) for every Raman excitation. The integration time of the CCD was 30 s for each spectral collection. Living cancer cells were randomly selected for RS data collection at room temperature, and each cell was measured once. No less than 30 isolated cells were measured for each group, and the background spectra surrounding the cells were acquired at the same time.

### 2.5. CCK-8 (MTT) Assay and Direct Cell Counting

The cholecystokinin (CCK-8) assay and direct cell counting are common methods to evaluate drug effects. Here, we used these methods to compare with RSI to verify the RSI method. The 50% concentration of inhibition (IC50) values from published data and our experimental results were used as reference drug doses [39,40]. For cytotoxicity testing, cancer cells were seeded in 96-well plates which had different gradient concentrations of drugs, and were incubated in order to create dose–response curve. Cells used for RS and CCK-8 assay were incubated under the same conditions. After incubation, cytotoxicity was measured with a CCK-8 kit (Sigma Aldrich, St. Louis, MO, USA) [12]. Optical density at 450 nm (OD450) was recorded with a Synergy HTX multi-mode reader (BioTek, Winooski, VT, USA). Then, direct cell counting was performed in sync with RS data collection using a hemocytometer. The cell counts were determined as the means of 8 cell groups from two gridded chambers of the hemocytometer.

### 2.6. In Vitro Growth Inhibition Assays

The MTT (3-[4,5-dimethylthiazol-2-yl]-2,5 diphenyl tetrazolium bromide) assay was used to measure cell death using a ratio of light absorption between experimental and control groups. Here, we defined the RS intensity ratios between experimental and control groups as a drug response. Meanwhile, we used a four-parameter dose–response (logistic) curve (4PL) to evaluate the RSI method. The model equation is:

(1) $$y=b+\frac{t-b}{1+10^{\left(Log\;IC50-x\right)\times h}},$$

where *x* is log-dose or concentration (log mol/L), and *y* is the response or decline in RS intensity or OD 450 for MTT. IC50 is the concentration of drug that gives a response halfway between the maximum and minimum responses. *h* is the Hill or slope factor (dimensionless), and *t* and *b* are the plateaus of the maximum and minimum responses (the maximal and minimal inhibition ratio from three independent assays), respectively.

### 2.7. Quantitative Measurements of the Heterogeneous Drug Responses

Principle Component Analysis (PCA) finds variables (components) accounting for as much as possible of the variance in multivariate data using. The largest possible variance between RSs of cancer cells were quantitatively calculated by using PCA. PCA uses eigenvalues and eigenvectors of variance-covariance or correlation matrices. Eigenvalues tell the variance accounting for corresponding eigenvectors (components). Full RS data for cancer cells within 450–1800 cm^−1^ was inputted as PCA variables for each test group, and PAST software [41] was used. An averaged heterogeneity coefficient *H* was defined as Equation (2):

(2) $$H=\frac{\sum_{i=n}PCE_{i}}{n},$$

where *n* is the cell number in the measurement group; *PCE_i_* is the eigenvalues of principal components. By calculating the *H* ratio (heterogeneity ratio) between drug-treated and control group cancer cell, we can obtain changes in heterogeneity of cancer cells after drug treatment.

### 2.8. Experimental Consistency Control

It is important to keep experimental condition consistency for drug sensitivity assays with the RSI method. Consistency mainly depends on the focus position on the cells with the laser beam, the laser power, and the stability of the Raman spectral setup. The RS system was standardized by measurement of the intensity and peak shift of the RS using a standard 5 µm polystyrene bead before each experiment.

The size of the spot of a Raman exciting laser beam on samples can be theoretically calculated by a Bassel function (~0.61λ/NA). This spot is about 520 nm in diameter, which is smaller than actual laser spot size. The size of the cancer cells in our experiment were ~(10–15) μm, as these cells had large nuclei. For RS measurements, the laser spot was focused on the cellular nucleus to avoid relative “position difference effects”. Thus, we created a stable RS curve and blocked organelle interference. Wavelength correction was carried out using a polystyrene bead prior to cell experiments too. For intensity corrections, the laser power before the objective and its relative position on the entrance slit of the spectrometer were held constant in all experiments. RSI fluctuation resulting from the bias of laser focus position on the cells was less than 3%, which was much less than the change caused by the drug (Figure S2 in Supporting Information). All these above-mentioned measures ensured that the RSI data reflected true cell activity.

### 2.9. Data Processing

RSI data processing was performed using a homemade software based on MATLAB (The MathWorks, Inc., Natick, MA, USA). Spectra were calibrated via the wavelength dependence of a standard 1001 cm^−1^ vibrational band of polystyrene beads before the RS measurements. For each spectrum, the background noise including the quartz contribution was removed by subtracting the background spectra from the raw spectral data. To do this and remove the effect due to instrument, the raw spectra data need to be normalized. In detail, we applied one inherent Raman peak of 413 cm^−1^ rooted from immersion oil in all measurements (including background RS) as an interior label, and all raw spectra were normalized by this peak. For every processed RS, the intensity of the main Raman peaks that corresponded to different chemical components related to cell death was extracted for drug response analyses. In addition, the area under the curve (AUC) of RS between 450–1800 cm^−1^, which represented the ensemble of various components within a cell, was obtained by RS curve integrals.

In this study, we used the changes both of the AUC and the single typical component peaks to evaluate drug effects. The biochemical phenomena related to cell apoptosis or oncosis are very complex. The Nomenclature Committee on Cell Death (NCCD) has proposed that specific biochemical analyses should not be employed as exclusive means to define apoptosis or oncosis, because different types of cell death can occur with different biochemical changes [42]. Moreover, the manner of cell death may obscure biochemical and functional heterogeneity. There may be distinct subtypes of one type of cell death which may be morphologically similar but are triggered through different biochemical routes [42,43,44]. The AUC contained all the changes in the components, which might result in a more accurate and suitable evaluation of the effects of drug treatments.

## 3. Results and Discussion

**Figure 1:**
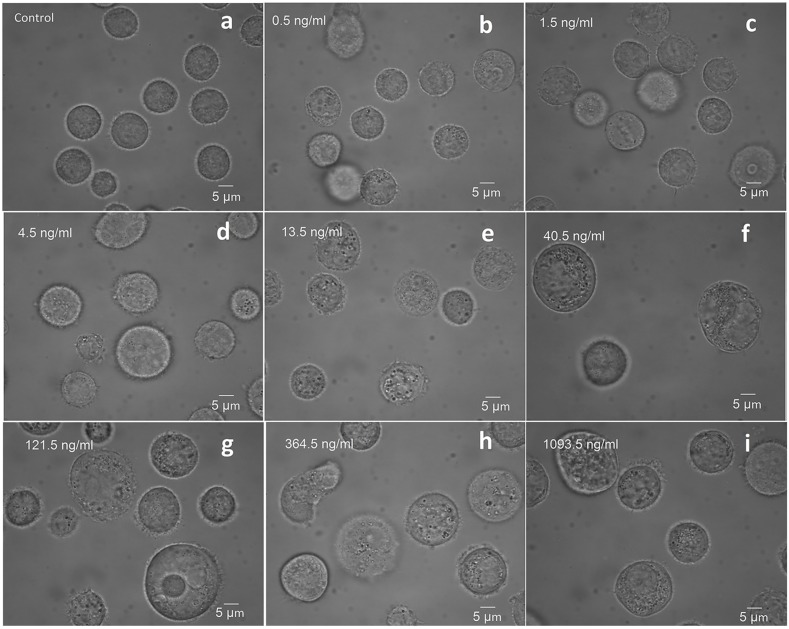
The bright field images of BGC823 cells under conditions of different concentrations of PTX treatment for 48 h. (**a**) Control group without PTX treatment; (**b**–**i**) The morphological change in BGC823 cells with increasing PTX concentrations. The cellular volume obviously increased with the drug concentration.

**Figure 2:**
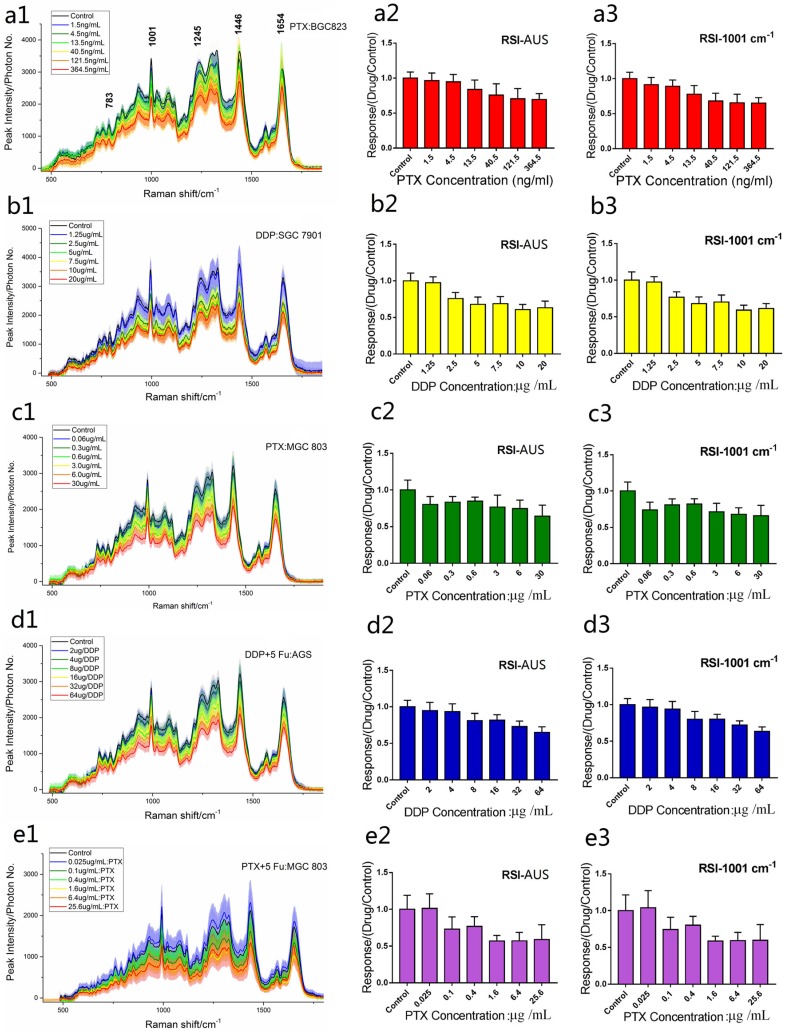
Dose–response relationships for different cancer cell lines with different anti-cancer drug treatments (expressed as drug(s): cell). (**a**–**e**), BGC823, SGC7901, MGC803, AGS, or MGC803 cells treated with concentration gradients of the drugs PTX, DDP, PTX, and combinations of DDP and 5 Fu, or PTX and 5 Fu, respectively; (**a1**–**e1**), the averaged RS from different drug concentrations; (**a2**–**e2**), the averaged RS area; (**a3**–**e3**), the 1001 cm^−1^ band; the averaged RSs and bar graphs present mean ± s.d., n ≥ 30 cells per condition. *p* < 0.001 for all groups (a one-way ANOVA).

The chemotherapy-induced cancer cell death that we observed had the typical morphological features of oncosis, including the fact that these cells became bigger than normal living cells, and cellular volume obviously increased with the drug concentration (Figure 1). The averaged Raman spectra for several cancer cells treated with different drugs are shown in Figure 2(a1–e1). The RS peaks correspond to molecular vibrations of cellular components, including nucleic acids, proteins, lipids, and carbohydrates. In eukaryotic cells, the 783 cm^−1^ band corresponds to the nucleic acid components of RNA or αDNA, namely, the uracil (U), thymine (T), and cytosine (C) ring vibrations. Therefore, major changes related to DNA can be observed at this peak [27,28]. Bands at 1001, 1245, and 1654 cm^−1^ can be attributed to proteins. The sharp band at 1001 cm^−1^ corresponds to a symmetric ring-breathing mode of phenylalanine [26,27], which is sensitive to cell death [25,45,46] and can be used for determining whether the cells are dead. The 1245 cm^−1^ band corresponds to amide III (bonds in β sheets), and the 1654 band cm^−1^ corresponds to amide I (α-helix), which are basic components of protein structure [18,47]. The 1446 cm^−1^ band corresponds to CH_2_ bending vibrations in proteins, carbohydrates, lipids, and DNA [25,27,28]. This demonstrates the single-cell RS intensity changes as a whole.

### 3.1. Chemotherapy Drug Dose-Effect Assay

To evaluate the utility of RSI, we measured drug effects for different cell lines. Figure 2 shows the changes in the mean RS of several cell lines treated with different drugs (Figure 2(a1–e1)) at 24 h, shown the change of their AUCs (Figure 2(a2–e2)), and a typical 1001 cm^−1^ band (Figure 2(a3–e3)) as drug concentration increases. The results showed that the RS peaks of drug-treated cells compared to the peaks form the control group decreased with increasing drug concentration, which suggests that the amount or density of nucleic acid, protein, and lipid could be lower during drug-induced cell death. This spectral presentation was consistent with the fact that the chromatin of the nucleolus and other cellular components is highly fragmented in the oncosis process as the drug concentration increases [25,26,27,28]. The quantitative change of the RSI-AUC and the 1001 cm^−1^ band from drug treated cancer cells express dose-response relationships. To verify the feasibility of RSI we compared RSI with MTT, the results are shown in Table 1, and the coefficient of determination, R^2^, was calculated from Equation (1). The mean coefficients of determination (computed by the least squares method) for the AUC were greater than that of RS-1001 cm^−1^ and lower than the MTT values. All R^2^ values were high, indicating that the MTT and RSI methods fitted the dose–response curves well.

Furthermore, regression analyses of the correlation coefficients between RSI and MTT, which express the correlation between RSI and MTT, were performed and the data are presented in Table 2. For the AUC and RSI-1001 cm^−1^, the correlation coefficients were highly consistent for all kind of cells and drugs, and for the AUC and MTT it was also consistent. The results indicate that both the AUC of RS and RAI-1001 can be used to evaluate drug sensitivity like MTT can. Meanwhile, we noticed that there are a few smaller correlation coefficients between the MTT and RSI methods, such as the case of the MGC803 cells treated with PTX, with a comparison between RSI-1001 cm^−1^ and MTT. This suggests that RSI is not exactly same as MTT in drug sensitivity assays because they have different evaluation criteria. Moreover, we observed that the difference between the RSI and MTT increased with drug dose. MTT was more sensitive at higher doses, due to the fact that the number of living cells decreased rapidly as drug concentration increased, while RSI evaluated the survival state of living cells.

### 3.2. Quantification of Heterogeneous Drug Responses

**Figure 3:**
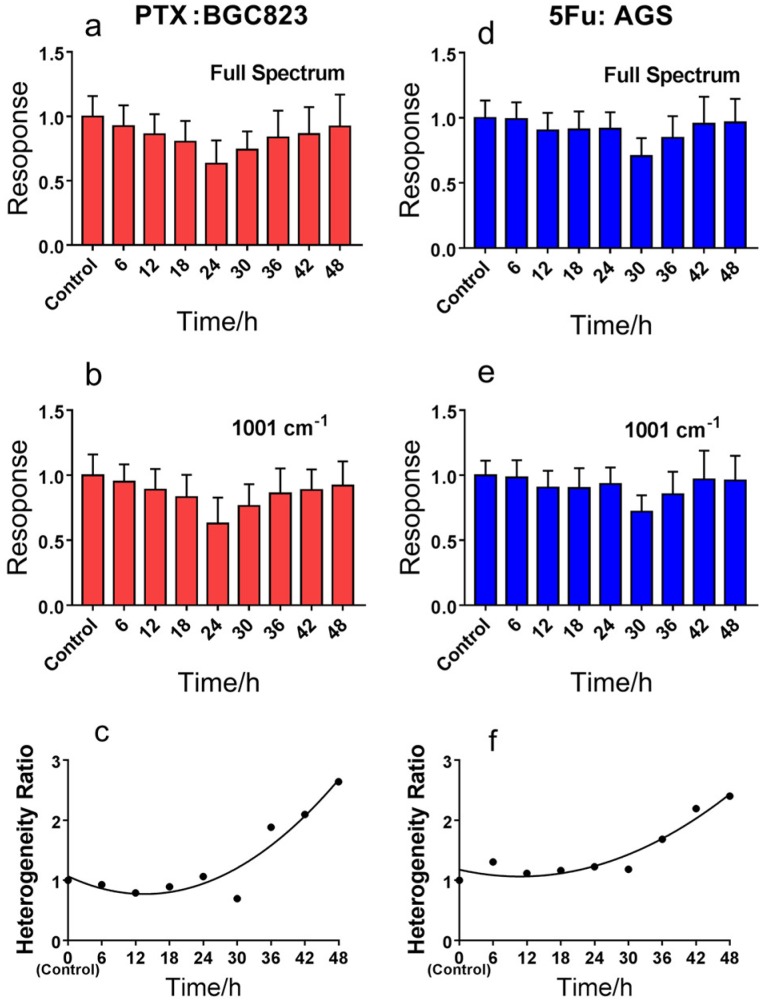
Drug response and heterogeneity changes for different cancer cell lines treated with anti-cancer drugs for multiple culture durations. (**a**,**b**), BGC 823 cells treated with 140 ng/mL PTX (~IC50). (**d**,**e**), AGS cells treated with 30 µg/mL PTX (~2 × IC50). Bars represent the mean ± s.d. (**c**,**f**), the heterogeneity ratio changes with drug treatment time for BGC 823 and AGS cells. The curve is a result of quadratic function fitting. Cell number was n ≥ 30 in each group. Data were collected every 6 h for 48 h.

We used the *H* ratio between drug-treated and control cells to describe the heterogeneous drug responses quantitatively. Figure 3 shows the RSI changes with cell drug exposure time for BGC823 cells treated with PTX and AGS cells treated with 5-FU. RS was collected every 6 h for 48 h. The results showed that cancer cells exposed to high concentrations of drugs for a long time became resistant. The numbers of living cells declined over 48 h due to drug-induced cell death (Figure S3 in Supporting Information). However, the RSI first decreased and then increased over the drug treatment time. For BGC823 cells, the RSI diminished initially and then increased after 24 h compared to controls. For AGS cells, likewise, the RSI became the lowest at 30 h. We think that the decrease in RSI resulted from drug-induced cell oncosis. Subsequent RSI increases were likely due to drug resistance of cells, because drug-sensitive cells were dead and were removed in the cell washing and centrifugation process. This was probably due to poor adaptation during long times of drug treatment, only the remaining drug-resistant cells were assayed with RSI. Other researchers have also reported similar results [27]. Since longer drug treatment times correlated to higher proportions of drug-resistant cell, the mean RSI increased with drug treatment time after 24 h. Moreover, the heterogeneity ratio between treated and control cells significantly increased with drug treatment time after 24 h for BGC823 and 30 h for AGS (Figure 3c,f), just as RSI increased. It follows that the heterogeneous characteristics of cancer cells became highlighted as drug-resistant characteristics increased.

### 3.3. Targeted Drug Growth/Inhibitor Evaluations

**Figure 4:**
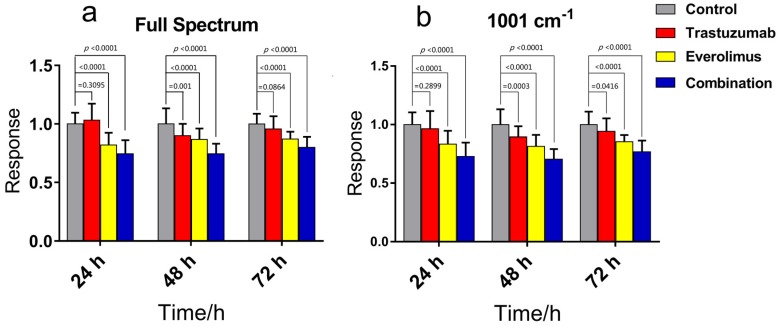
The RSI method to measure molecularly targeted anti-cancer drug effects. Trastuzumab (250 nM) and everolimus (20 nM, referring to the plasma concentration) were used singly or together. Bars represent mean ± s.d. of drug treated cells to control cells, n ≥ 30 cells per group. Data were collected at 24, 48, and 72 h. *p* value: two-tailed Student’s *t*-test.

In order to evaluate the feasibility of RSI applied in the targeted drug sensitivity assay, trastuzumab and everolimus were applied to NCI-N87 cells. Bright field images showed no significant difference in morphological features between treated and control groups for either of the targeted drugs, but there was a significant difference in RSI. This indicates that cell morphological change measurements are not reliable for evaluating effects of targeted anti-cancer drugs, especially for drug induced non-oncosis cell death. Figure 4 shows the RSI measurement results for NCI-N87 cells treated with trastuzumab or everolimus alone and in combination for 24, 48, and 72 h. The response of the cells treated with trastuzumab was not significantly different at 24 and 72 h, but at 48 h, it had a significant difference between treated and control groups. While for NCI-N87 cells treated with everolimus alone or in combination, the responses of the treated groups were significantly different from controls at all time points (*p* < 0.0001). In addition, in comparison to trastuzumab, everolimus has a significantly inhibitory activity on the proliferation of NCI-N87 cells, although the inhibitory effects were the most obvious for the combination of the two drugs. These results are in agreement with published data [48,49].

**Figure 5:**
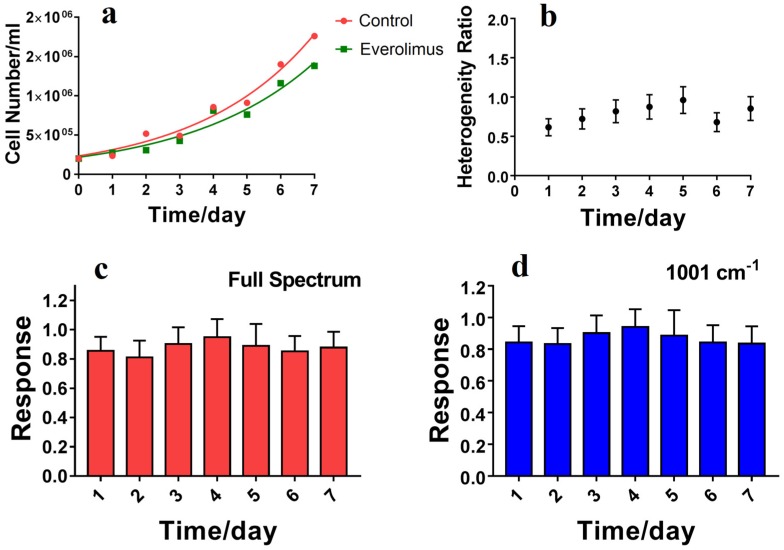
The long-term inhibitory effect and changes in heterogeneous characteristics due to everolimus treatment of NCI-N87. (**a**) Exponential fittings of proliferation rates of NCI-N87 cells. The rate constant for the control group was 0.2904 and the doubling-time was 2.387 days, with R^2^ = 0.9785. The rate constant for the everolimus group was 0.2684, with a doubling-time of 2.853 days and R^2^ = 0.9622; (**b**) Changes in the heterogeneous characteristics of NCI-N87 cells with the everolimus treatment time. The s.d. of the heterogeneity obtained from control groups over 7 days; (**c**) The drug response of NCI-N87 cells with everolimus treatment (RSI-AUC); (**d**) The drug response of NCI-N87 cells with everolimus treatment (RSI-1001 cm^−1^). Bars represent the mean ± s.d. of drug treated cells to control cells, n ≥ 30.

The long-term inhibitory effect of everolimus and heterogeneous characteristics of NCI-N87 were studied using RSI. NCI-N87 cells were treated with 20 nM everolimus, and data were collected every 24 h for 7 days (See Figure 5). In spite of the fact that the cell number grew with time, the proliferation rates of NCI-N87 cells in the everolimus group was clearly lower than in the control group (Figure 5a), which indicated that the targeted drug had significant inhibitory effects. However, this indicated that unlike chemotherapeutics, the targeted drug did not induce cancer cell death but inhibited cancer cell proliferation. Moreover, RSI also showed that everolimus significantly inhibited the growth of NCI-N87 cells compared to controls (Figure 5c,d). Compare to the cytotoxic drug treatment (Figure 3c,f), the heterogeneous ratio fluctuations were not obvious over the 7 days (Figure 5b), illustrating that molecularly targeted drugs have less possibility to induce drug resistance.

## 4. Conclusions

We proposed a drug sensitivity testing method using RSI which is consistent with conventional DST methods, such as MTT and direct cell counting. The RSI method can overcome the limitations of conventional DST methods, such as being unsuitable for targeted drugs. RSI can provide a new method for quantifying the heterogeneity of cancer cell responses to anti-cancer drugs. RSI gives a possible approach for molecularly targeted anti-cancer drug sensitivity testing by measuring cell viability. This research shows that label-free RSI is a sensitive and multi-functional method for anti-cancer drug sensitivity.

## Figures and Tables

**Table 1:** Coefficient of determination (R^2^) for RSI and MTT fitting.

Test Groups	AUC	RSI-1001 cm^−1^	CCK-8 (MTT)
PTX: BGC 823	0.9960	0.9995	0.9726
DDP: SGC 7901	0.9721	0.9518	0.9961
PTX: MGC 803	0.9424	0.8528	0.9761
DDP + 5-Fu: AGS	0.9531	0.9540	0.9949
PTX + 5-Fu: MGC 803	0.9009	0.9009	0.8838
Mean R^2^	0.9529 ± 0.0355	0.9315 ± 0.0565	0.9647 ± 0.0465

**Table 2:** Regression analyses for correlation coefficients between the RSI and MTT methods.

Test Groups	AUC: MTT	RSI-1001 cm^−1^:MTT	AUC:RSI-1001 cm^−1^
PTX: BGC823	0.9267 (*p* ^1^ = 0.008)	0.9051 (*p* = 0.013)	0.9956 (*p* < 0.000)
DDP: SGC7901	0.7352 (*p* = 0.096)	0.7713 (*p* = 0.072)	0.9961 (*p* < 0.000)
PTX: MGC803	0.9316 (*p* = 0.007)	0.6963 (*p* = 0.124)	0.9021 (*p* = 0.014)
DDP + 5Fu: AGS	0.8832 (*p* = 0.019)	0.8705 (*p* = 0.024)	0.9986 (*p* < 0.000)
PTX + 5Fu: MGC803	0.8024 (*p* = 0.054)	0.8171 (*p* = 0.047)	0.9992 (*p* < 0.000)

^1^*p* value: the two-tailed probabilities that two testing results were uncorrelated.

## Supplementary Materials

Supplementary materials are available online: Figure S1: Configuration of the Raman spectroscopy. A single laser beam at the wavelength of 532 nm is directed to the microscope via dichroic mirrors (D1) and coupled into the objective with free working distance of 0.29 mm. The back scattering Raman light is collected by the same objective and then delivered into the spectrometer via a dichroic mirror (D2). A pinhole with a diameter of 100 μm was used to create a confocal system. A notch filter is placed before the entrance of the spectrometer to filter the Rayleigh scattered light from the sample. The RS is finally recorded with a liquid nitrogen (LN)-cooled spectroscopic CCD, Figure S2: The focusing position controls under a bright light view of the cell. The axial position can be controlled with ~1 μm precision by observing the edge of the cell under a bright light view. When focusing on the center of the cell accurately, a bright and fine ring is seen on the edge of the cell due to the bright light. The ring would become dark and wide when out of focus. (a–e), The axial focusing position from 2 μm above the cell center to 2 μm below the cell center. (f) The RS corresponding to (a–e). The differences in the area under the curves and between lines at 0 μm and ±1 μm were less than 2.8%. This is less than the change caused by the drug, Figure S3: Cell counts of cancer cells over drug treatment every 6 h for 48 h. (a) BGC 823 cells under 140 ng/mL PTX treatment. (b) AGS cells under 30 μg/mL PTX treatment.
