# Supplementary material for: Anti-Cancer Drug Sensitivity Assay with Quantitative Heterogeneity Testing Using Single-Cell Raman Spectroscopy
Source: Molecules. 2018 Nov 7;23(11):2903. doi: 10.3390/molecules23112903 (PMC6278387; doi:10.3390/molecules23112903)
Supplement: Supplementary file 1 [file molecules-23-02903-s001.pdf]

Supplementary materials

Anti-Cancer Drug Sensitivity Assay with Quantitative Heterogeneity Testing using Single-Cell Raman Spectroscopy

Yong Zhang [*,1,2], *Jingjing Xu*[3], *Yuezhou Yu*[3], *Wenhao Shang*[3], *and Anpei Ye*[1,3]

[1] Key Laboratory for the Physics and Chemistry of Nanodevices, School of Electronics Engineering and Computer Science, Peking University, No.5 Yiheyuan Road, 100871 Beijing, China

[2] Beijing Institute of Biomedicine, No.15 Xinjiangongmen Road，100091 Beijing, China

[3] Academy for Advanced Interdisciplinary Studies, Peking University, No.5 Yiheyuan Road, 100871 Beijing, China；

Supplementary Figures

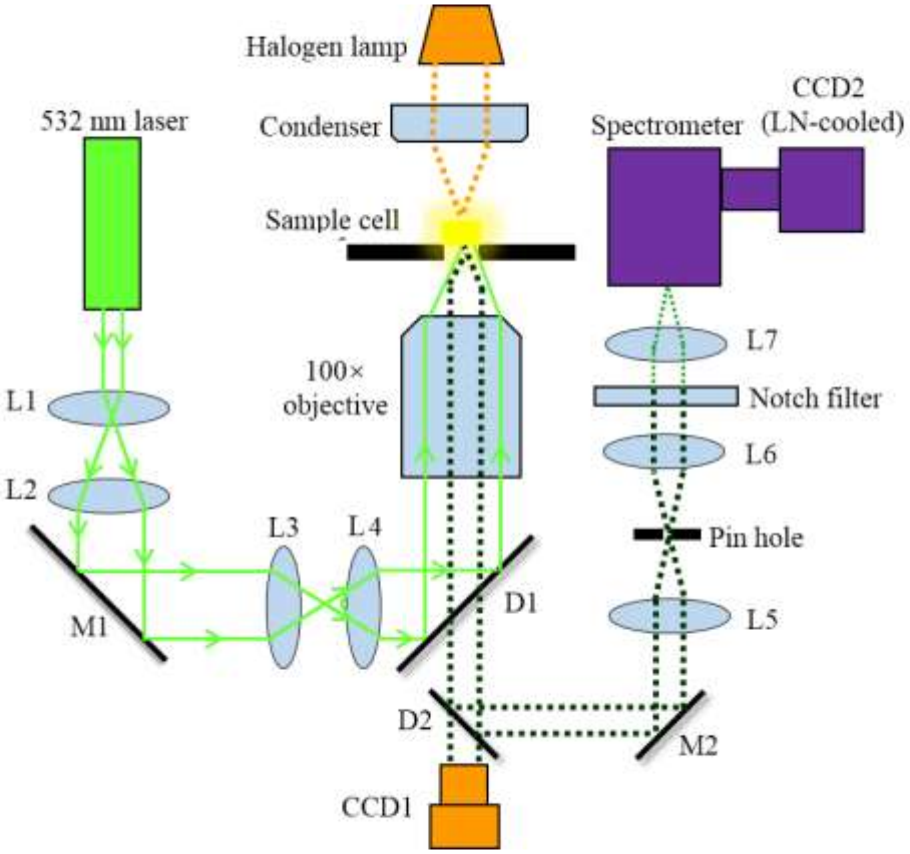

Figure S1. Configuration of the Raman spectroscopy. A single laser beam at the wavelength of 532 nm is directed to the microscope *via* dichroic mirrors (D1) and coupled into the objective with free working distance of 0.29 mm. The back scattering Raman light is collected by the same objective and then delivered into the spectrometer *via* a dichroic mirror (D2). A pinhole with a diameter of 100 μm was used to create a confocal system. A notch filter is placed before the entrance of the spectrometer to filter the Rayleigh scattered light from the sample. The RS is finally recorded with a liquid nitrogen (LN)-cooled spectroscopic CCD.

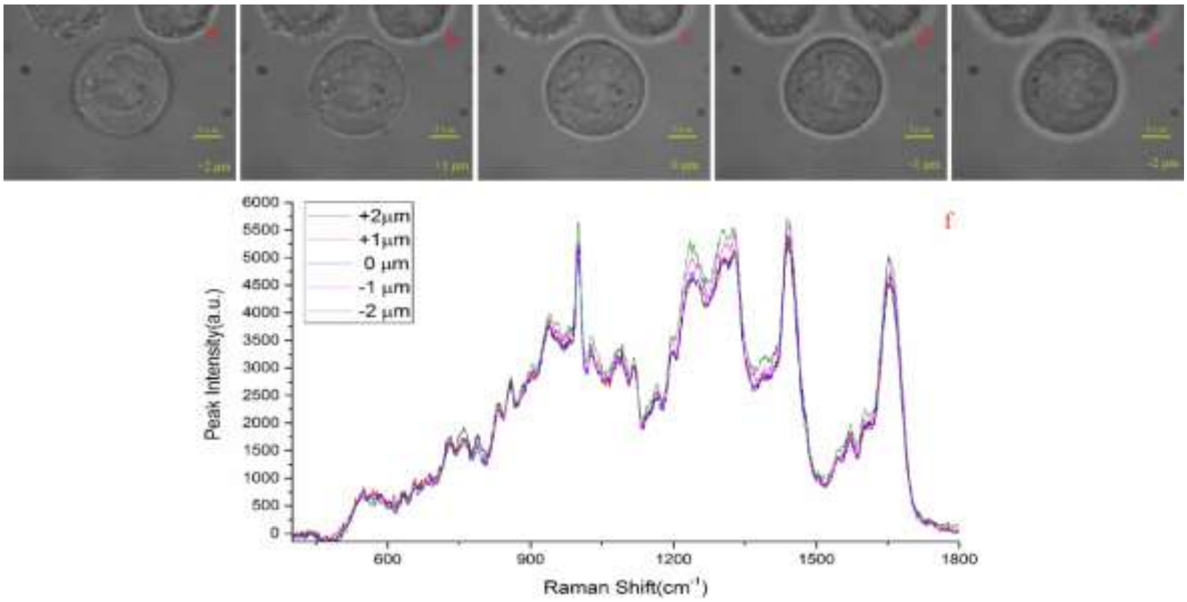

Figure S2. The focusing position controls under a bright light view of the cell. The axial position can be controlled with ~1µm precision by observing the edge of the cell under a bright light view. When focusing on the center of the cell accurately, a bright and fine ring is seen on the edge of the cell due to the bright light. The ring would become dark and wide when out of focus. (a)-(e), The axial focusing position from 2 µm above the cell center to 2 µm below the cell center. (f) The RS corresponding to (a)-(e). The differences in the area under the curves and between lines at 0 µm and ±1 µm were less than 2.8%. This is less than the change caused by the drug.

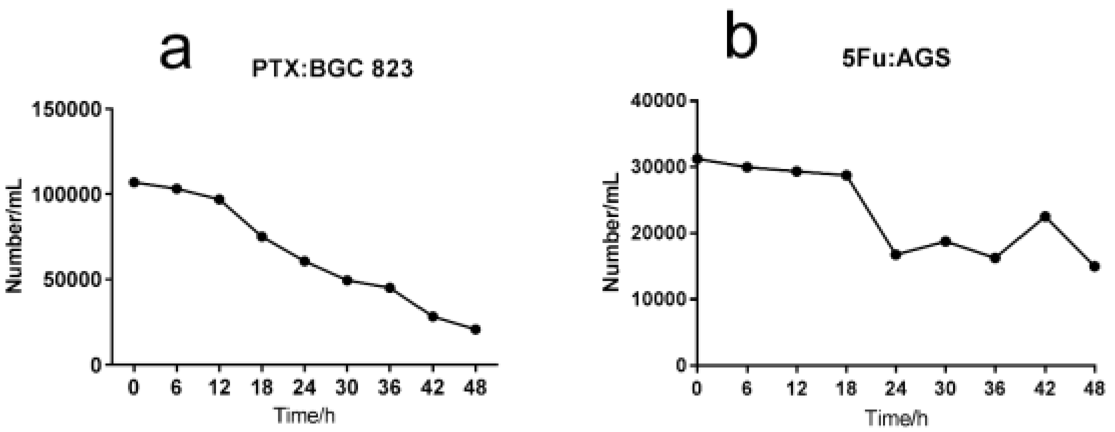

Figure S3. Cell counts of cancer cells over drug treatment every 6 h for 48 h. (a) BGC 823 cells under 140ng/ml PTX treatment.(b) AGS cells under 30 ug/ml PTX treatment.
